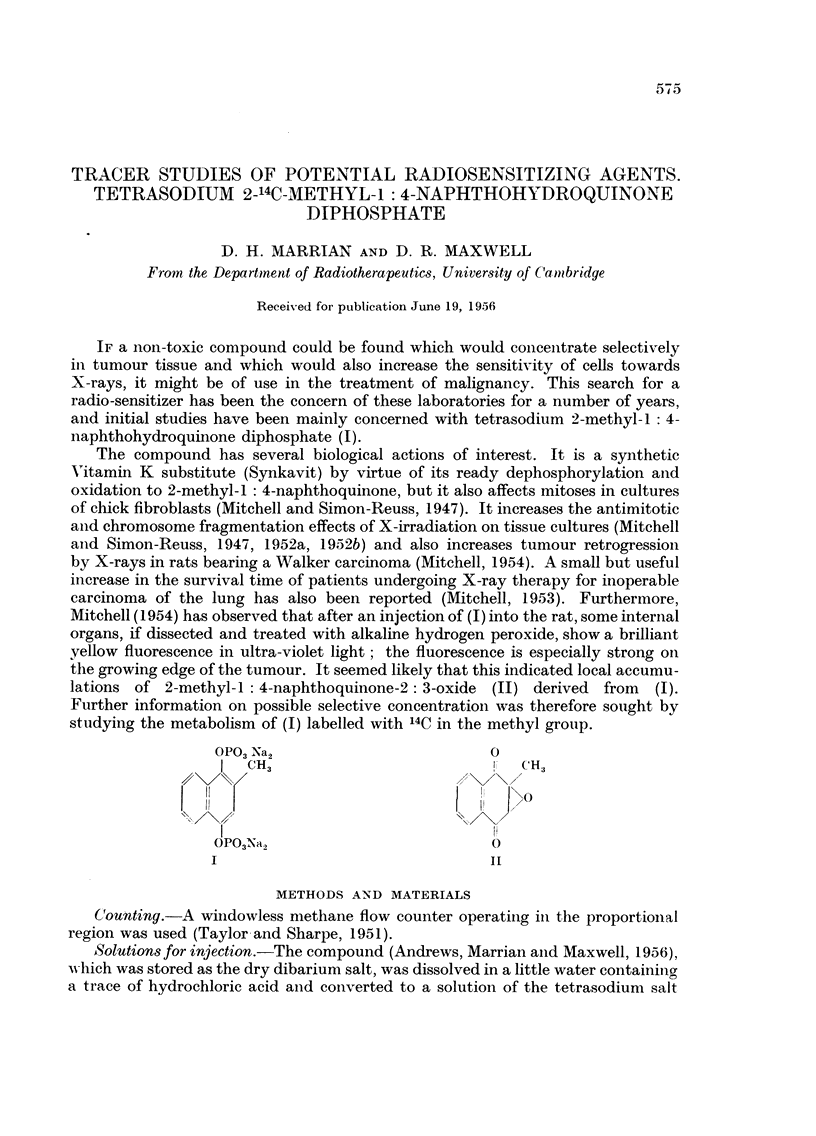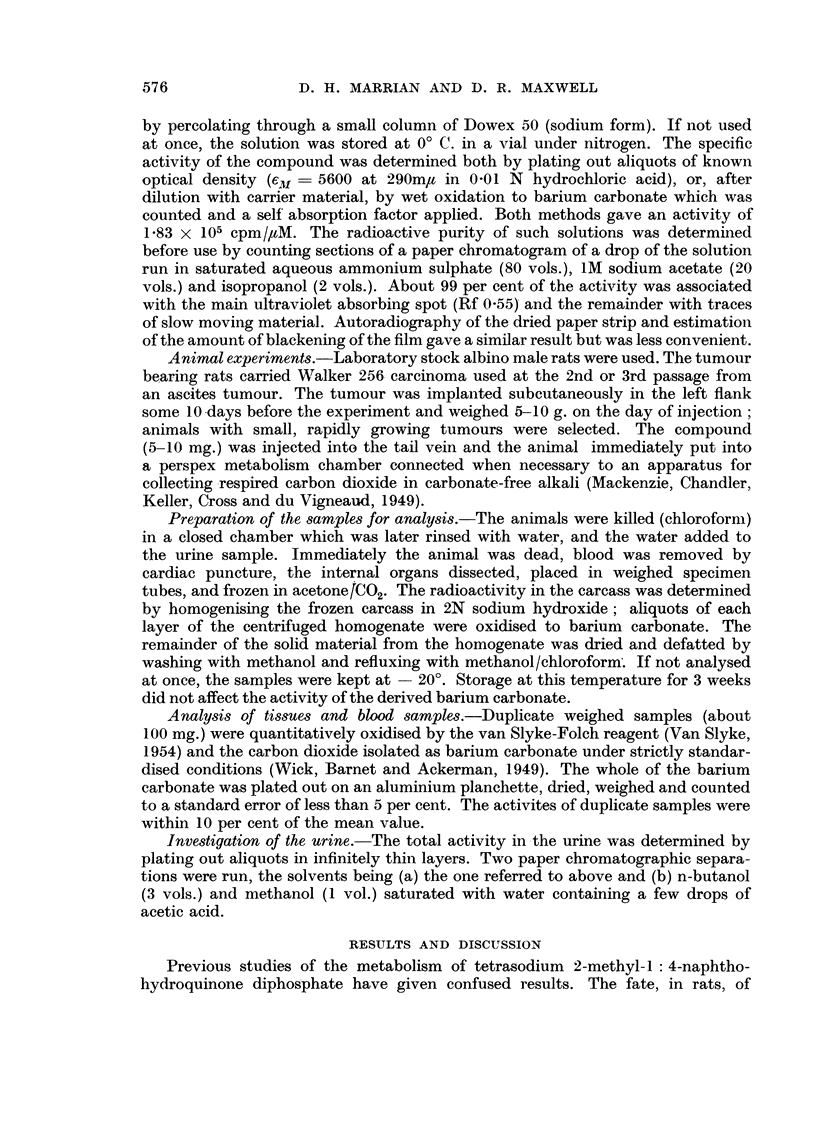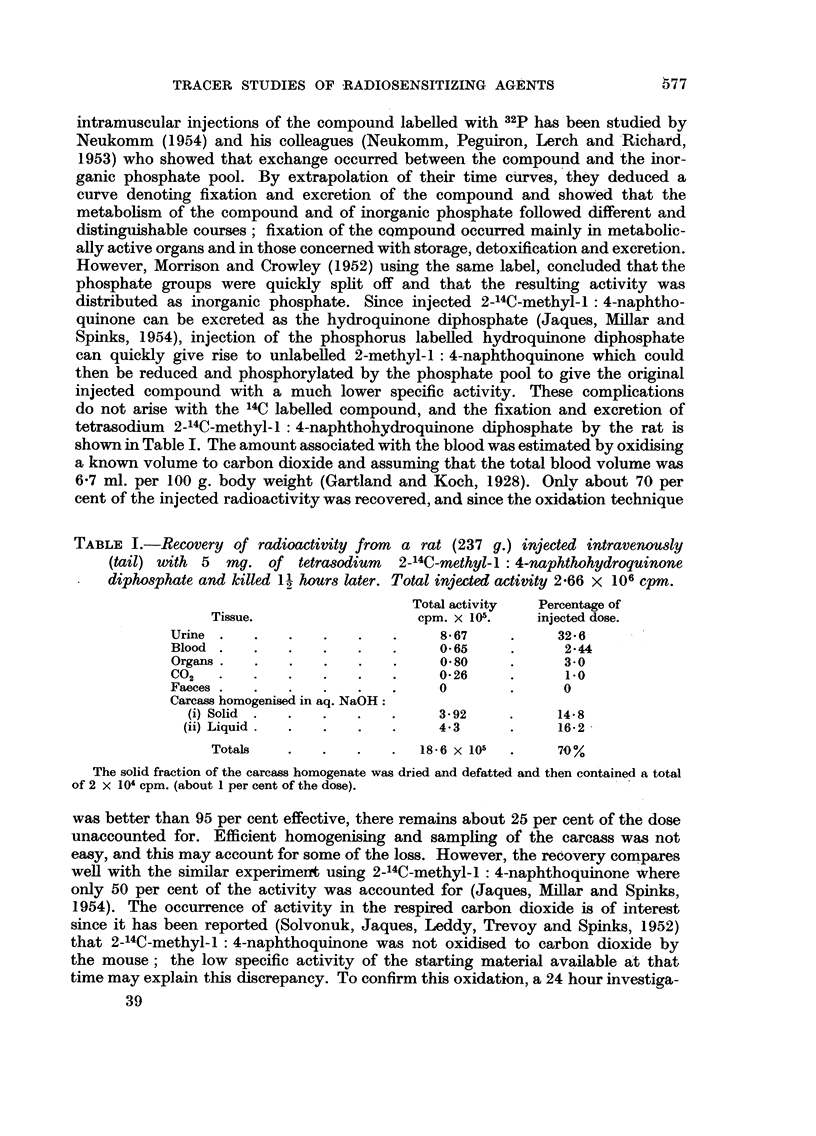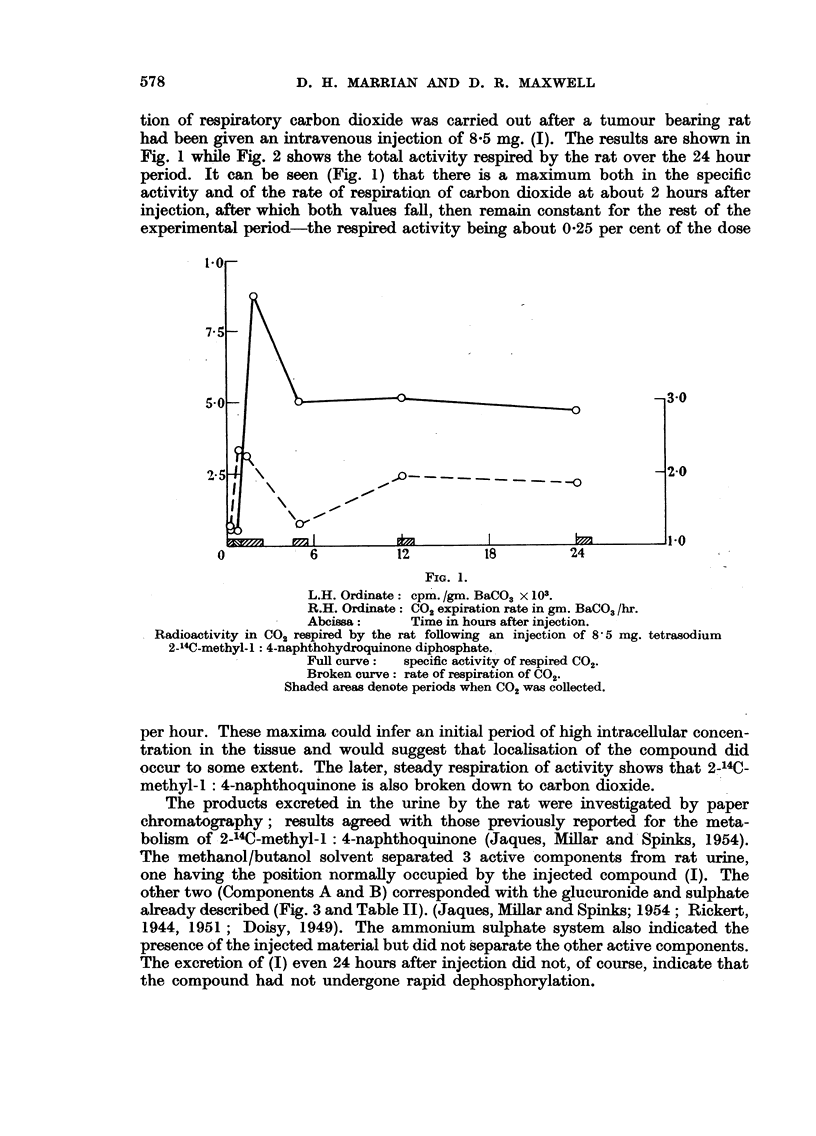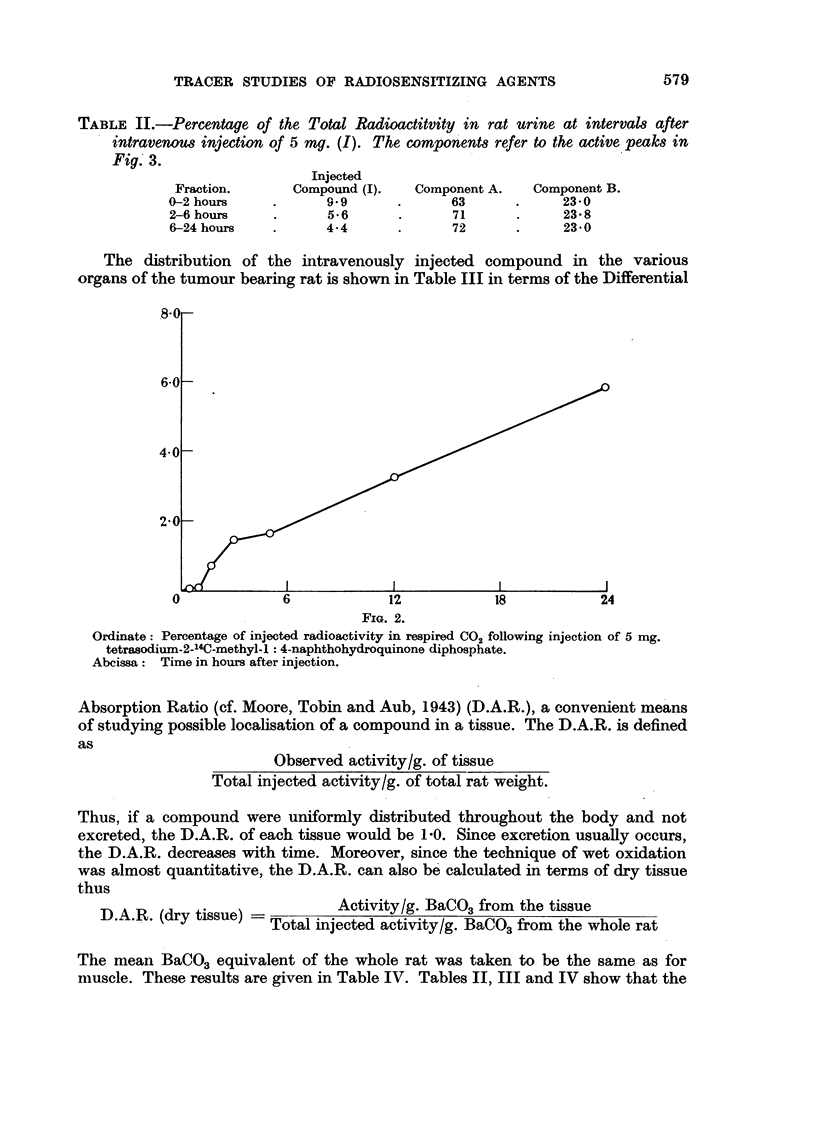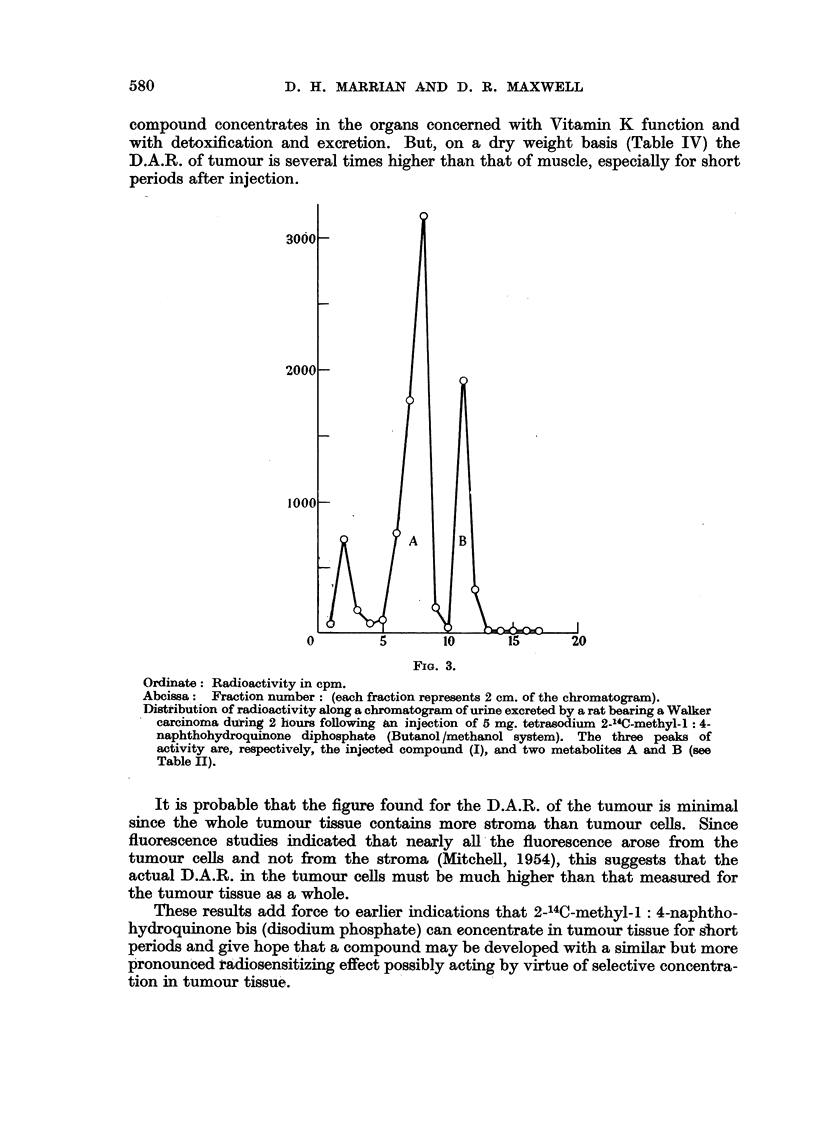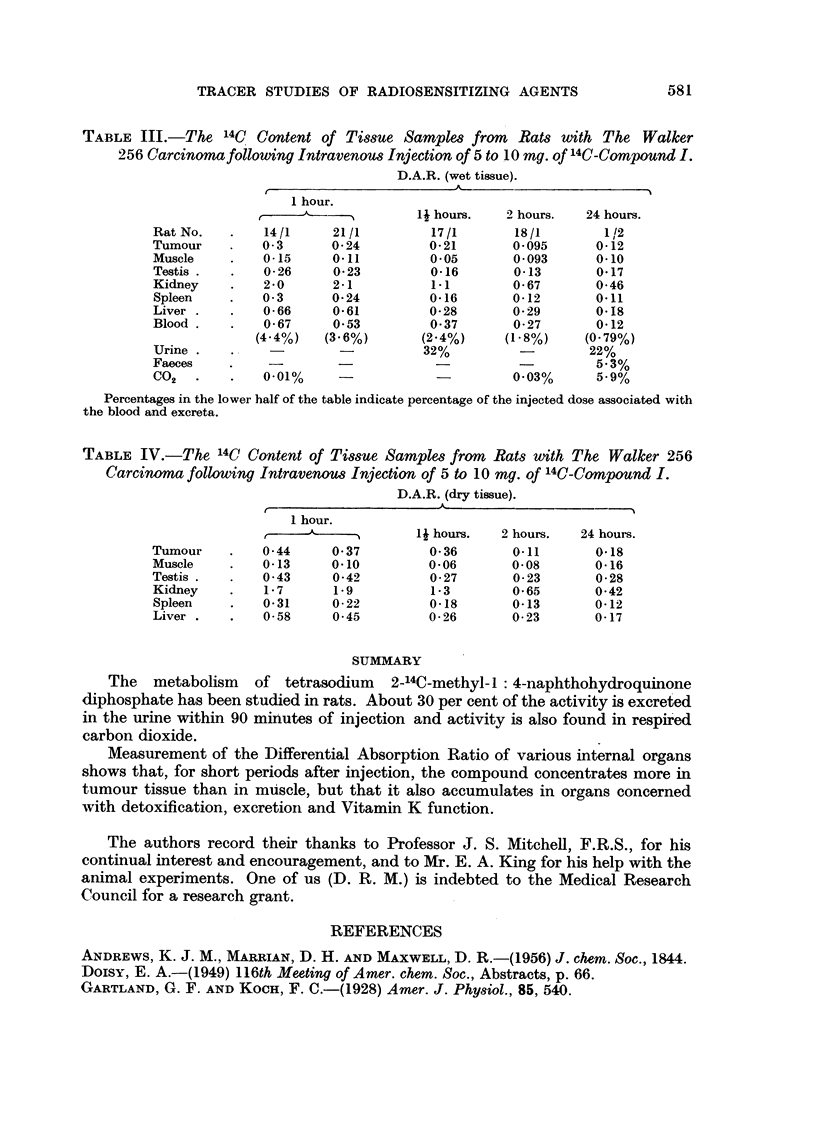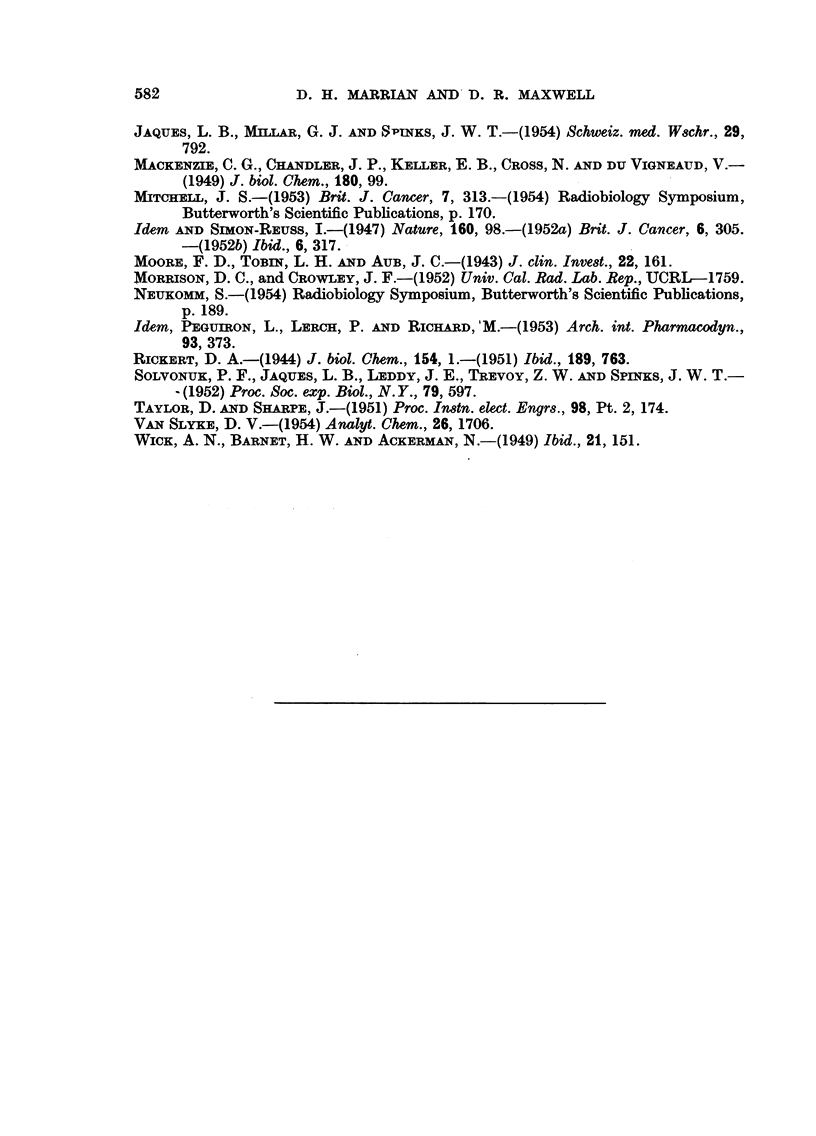# Tracer Studies of Potential Radiosensitizing Agents. Tetrasodium 2-^14^C-Methyl-1:4-Naphthohydroquinone Diphosphate

**DOI:** 10.1038/bjc.1956.69

**Published:** 1956-09

**Authors:** D. H. Marrian, D. R. Maxwell


### TRACER STUDIES OF POTENTIAL RADIOSENSITIZING AGENTS. TETRASODIUM 2-$^{14}$C-METHYL-1 : 4-NAPHTHOHYDROQUINONE DIPHOSPHATE

D. H. MARRIAN AND D. R. MAXWELL

*From the Department of Radiotherapeutics, University of Cambridge*

Received for publication June 19, 1956

If a non-toxic compound could be found which would concentrate selectively in tumour tissue and which would also increase the sensitivity of cells towards X-rays, it might be of use in the treatment of malignancy. This search for a radio-sensitizer has been the concern of these laboratories for a number of years, and initial studies have been mainly concerned with tetrasodium 2-methyl-1 : 4-naphthohydroquinone diphosphate (I).

The compound has several biological actions of interest. It is a synthetic Vitamin K substitute (Synkavit) by virtue of its ready dephosphorylation and oxidation to 2-methyl-1 : 4-naphthoquinone, but it also affects mitoses in cultures of chick fibroblasts (Mitchell and Simon-Reuss, 1947). It increases the antimitotic and chromosome fragmentation effects of X-irradiation on tissue cultures (Mitchell and Simon-Reuss, 1947, 1952a, 1952*b*) and also increases tumour retrogression by X-rays in rats bearing a Walker carcinoma (Mitchell, 1954). A small but useful increase in the survival time of patients undergoing X-ray therapy for inoperable carcinoma of the lung has also been reported (Mitchell, 1953). Furthermore, Mitchell (1954) has observed that after an injection of (I) into the rat, some internal organs, if dissected and treated with alkaline hydrogen peroxide, show a brilliant yellow fluorescence in ultra-violet light ; the fluorescence is especially strong on the growing edge of the tumour. It seemed likely that this indicated local accumulations of 2-methyl-1 : 4-naphthoquinone-2 : 3-oxide (II) derived from (I). Further information on possible selective concentration was therefore sought by studying the metabolism of (I) labelled with $^{14}$C in the methyl group.

I

II

METHODS AND MATERIALS

*Counting.*—A windowless methane flow counter operating in the proportional region was used (Taylor and Sharpe, 1951).

*Solutions for injection.*—The compound (Andrews, Marrian and Maxwell, 1956), which was stored as the dry dibarium salt, was dissolved in a little water containing a trace of hydrochloric acid and converted to a solution of the tetrasodium salt by percolating through a small column of Dowex 50 (sodium form). If not used at once, the solution was stored at 0° C. in a vial under nitrogen. The specific activity of the compound was determined both by plating out aliquots of known optical density ($\epsilon_M = 5600$ at 290m$\mu$ in 0·01 N hydrochloric acid), or, after dilution with carrier material, by wet oxidation to barium carbonate which was counted and a self absorption factor applied. Both methods gave an activity of $1 \cdot 83 \times 10^5$ cpm/$\mu$M. The radioactive purity of such solutions was determined before use by counting sections of a paper chromatogram of a drop of the solution run in saturated aqueous ammonium sulphate (80 vols.), 1M sodium acetate (20 vols.) and isopropanol (2 vols.). About 99 per cent of the activity was associated with the main ultraviolet absorbing spot (Rf 0·55) and the remainder with traces of slow moving material. Autoradiography of the dried paper strip and estimation of the amount of blackening of the film gave a similar result but was less convenient.

*Animal experiments.*—Laboratory stock albino male rats were used. The tumour bearing rats carried Walker 256 carcinoma used at the 2nd or 3rd passage from an ascites tumour. The tumour was implanted subcutaneously in the left flank some 10 days before the experiment and weighed 5–10 g. on the day of injection ; animals with small, rapidly growing tumours were selected. The compound (5–10 mg.) was injected into the tail vein and the animal immediately put into a perspex metabolism chamber connected when necessary to an apparatus for collecting respired carbon dioxide in carbonate-free alkali (Mackenzie, Chandler, Keller, Cross and du Vigneaud, 1949).

*Preparation of the samples for analysis.*—The animals were killed (chloroform) in a closed chamber which was later rinsed with water, and the water added to the urine sample. Immediately the animal was dead, blood was removed by cardiac puncture, the internal organs dissected, placed in weighed specimen tubes, and frozen in acetone/$CO_2$. The radioactivity in the carcass was determined by homogenising the frozen carcass in 2N sodium hydroxide ; aliquots of each layer of the centrifuged homogenate were oxidised to barium carbonate. The remainder of the solid material from the homogenate was dried and defatted by washing with methanol and refluxing with methanol/chloroform. If not analysed at once, the samples were kept at − 20°. Storage at this temperature for 3 weeks did not affect the activity of the derived barium carbonate.

*Analysis of tissues and blood samples.*—Duplicate weighed samples (about 100 mg.) were quantitatively oxidised by the van Slyke-Folch reagent (Van Slyke, 1954) and the carbon dioxide isolated as barium carbonate under strictly standardised conditions (Wick, Barnet and Ackerman, 1949). The whole of the barium carbonate was plated out on an aluminium planchette, dried, weighed and counted to a standard error of less than 5 per cent. The activites of duplicate samples were within 10 per cent of the mean value.

*Investigation of the urine.*—The total activity in the urine was determined by plating out aliquots in infinitely thin layers. Two paper chromatographic separations were run, the solvents being (a) the one referred to above and (b) n-butanol (3 vols.) and methanol (1 vol.) saturated with water containing a few drops of acetic acid.

### RESULTS AND DISCUSSION

Previous studies of the metabolism of tetrasodium 2-methyl-1 : 4-naphthohydroquinone diphosphate have given confused results. The fate, in rats, of intramuscular injections of the compound labelled with $^{32}P$ has been studied by Neukomm (1954) and his colleagues (Neukomm, Peguiron, Lerch and Richard, 1953) who showed that exchange occurred between the compound and the inorganic phosphate pool. By extrapolation of their time curves, they deduced a curve denoting fixation and excretion of the compound and showed that the metabolism of the compound and of inorganic phosphate followed different and distinguishable courses ; fixation of the compound occurred mainly in metabolically active organs and in those concerned with storage, detoxification and excretion. However, Morrison and Crowley (1952) using the same label, concluded that the phosphate groups were quickly split off and that the resulting activity was distributed as inorganic phosphate. Since injected 2-$^{14}C$-methyl-1 : 4-naphthoquinone can be excreted as the hydroquinone diphosphate (Jaques, Millar and Spinks, 1954), injection of the phosphorus labelled hydroquinone diphosphate can quickly give rise to unlabelled 2-methyl-1 : 4-naphthoquinone which could then be reduced and phosphorylated by the phosphate pool to give the original injected compound with a much lower specific activity. These complications do not arise with the $^{14}C$ labelled compound, and the fixation and excretion of tetrasodium 2-$^{14}C$-methyl-1 : 4-naphthohydroquinone diphosphate by the rat is shown in Table I. The amount associated with the blood was estimated by oxidising a known volume to carbon dioxide and assuming that the total blood volume was 6·7 ml. per 100 g. body weight (Gartland and Koch, 1928). Only about 70 per cent of the injected radioactivity was recovered, and since the oxidation technique

TABLE I.—*Recovery of radioactivity from a rat (237 g.) injected intravenously (tail) with 5 mg. of tetrasodium 2-$^{14}C$-methyl-1 : 4-naphthohydroquinone diphosphate and killed $1\frac{1}{2}$ hours later. Total injected activity $2·66 \times 10^6$ cpm.*

| Tissue. | Total activity cpm. $\times 10^5$. | Percentage of injected dose. |
|---|---|---|
| Urine | 8·67 | 32·6 |
| Blood | 0·65 | 2·44 |
| Organs | 0·80 | 3·0 |
| $CO_2$ | 0·26 | 1·0 |
| Faeces | 0 | 0 |
| Carcass homogenised in aq. NaOH : | | |
| (i) Solid | 3·92 | 14·8 |
| (ii) Liquid | 4·3 | 16·2 |
| Totals | $18·6 \times 10^5$ | 70% |

The solid fraction of the carcass homogenate was dried and defatted and then contained a total of $2 \times 10^4$ cpm. (about 1 per cent of the dose).

was better than 95 per cent effective, there remains about 25 per cent of the dose unaccounted for. Efficient homogenising and sampling of the carcass was not easy, and this may account for some of the loss. However, the recovery compares well with the similar experiment using 2-$^{14}C$-methyl-1 : 4-naphthoquinone where only 50 per cent of the activity was accounted for (Jaques, Millar and Spinks, 1954). The occurrence of activity in the respired carbon dioxide is of interest since it has been reported (Solvonuk, Jaques, Leddy, Trevoy and Spinks, 1952) that 2-$^{14}C$-methyl-1 : 4-naphthoquinone was not oxidised to carbon dioxide by the mouse ; the low specific activity of the starting material available at that time may explain this discrepancy. To confirm this oxidation, a 24 hour investigation of respiratory carbon dioxide was carried out after a tumour bearing rat had been given an intravenous injection of 8·5 mg. (I). The results are shown in Fig. 1 while Fig. 2 shows the total activity respired by the rat over the 24 hour period. It can be seen (Fig. 1) that there is a maximum both in the specific activity and of the rate of respiration of carbon dioxide at about 2 hours after injection, after which both values fall, then remain constant for the rest of the experimental period—the respired activity being about 0·25 per cent of the dose

FIG. 1.

L.H. Ordinate : cpm./gm. $BaCO_3 \times 10^3$.
R.H. Ordinate : $CO_2$ expiration rate in gm. $BaCO_3$/hr.
Abcissa : Time in hours after injection.
Radioactivity in $CO_2$ respired by the rat following an injection of 8·5 mg. tetrasodium 2-$^{14}$C-methyl-1 : 4-naphthohydroquinone diphosphate.
Full curve : specific activity of respired $CO_2$.
Broken curve : rate of respiration of $CO_2$.
Shaded areas denote periods when $CO_2$ was collected.

per hour. These maxima could infer an initial period of high intracellular concentration in the tissue and would suggest that localisation of the compound did occur to some extent. The later, steady respiration of activity shows that 2-$^{14}$C-methyl-1 : 4-naphthoquinone is also broken down to carbon dioxide.

The products excreted in the urine by the rat were investigated by paper chromatography ; results agreed with those previously reported for the metabolism of 2-$^{14}$C-methyl-1 : 4-naphthoquinone (Jaques, Millar and Spinks, 1954). The methanol/butanol solvent separated 3 active components from rat urine, one having the position normally occupied by the injected compound (I). The other two (Components A and B) corresponded with the glucuronide and sulphate already described (Fig. 3 and Table II). (Jaques, Millar and Spinks; 1954 ; Rickert, 1944, 1951 ; Doisy, 1949). The ammonium sulphate system also indicated the presence of the injected material but did not separate the other active components. The excretion of (I) even 24 hours after injection did not, of course, indicate that the compound had not undergone rapid dephosphorylation.

TABLE II.—*Percentage of the Total Radioactitvity in rat urine at intervals after intravenous injection of* 5 *mg.* (*I*). *The components refer to the active peaks in Fig.* 3.

| Fraction. | Injected Compound (I). | Component A. | Component B. |
|---|---|---|---|
| 0–2 hours | 9·9 | 63 | 23·0 |
| 2–6 hours | 5·6 | 71 | 23·8 |
| 6–24 hours | 4·4 | 72 | 23·0 |

The distribution of the intravenously injected compound in the various organs of the tumour bearing rat is shown in Table III in terms of the Differential

FIG. 2.

Ordinate : Percentage of injected radioactivity in respired $CO_2$ following injection of 5 mg. tetrasodium-2-$^{14}$C-methyl-1 : 4-naphthohydroquinone diphosphate.
Abcissa : Time in hours after injection.

Absorption Ratio (cf. Moore, Tobin and Aub, 1943) (D.A.R.), a convenient means of studying possible localisation of a compound in a tissue. The D.A.R. is defined as

$$\frac{\text{Observed activity/g. of tissue}}{\text{Total injected activity/g. of total rat weight.}}$$

Thus, if a compound were uniformly distributed throughout the body and not excreted, the D.A.R. of each tissue would be 1·0. Since excretion usually occurs, the D.A.R. decreases with time. Moreover, since the technique of wet oxidation was almost quantitative, the D.A.R. can also be calculated in terms of dry tissue thus

$$\text{D.A.R. (dry tissue)} = \frac{\text{Activity/g. } BaCO_3 \text{ from the tissue}}{\text{Total injected activity/g. } BaCO_3 \text{ from the whole rat}}$$

The mean $BaCO_3$ equivalent of the whole rat was taken to be the same as for muscle. These results are given in Table IV. Tables II, III and IV show that the compound concentrates in the organs concerned with Vitamin K function and with detoxification and excretion. But, on a dry weight basis (Table IV) the D.A.R. of tumour is several times higher than that of muscle, especially for short periods after injection.

FIG. 3.

Ordinate : Radioactivity in cpm.
Abcissa : Fraction number : (each fraction represents 2 cm. of the chromatogram).
Distribution of radioactivity along a chromatogram of urine excreted by a rat bearing a Walker carcinoma during 2 hours following an injection of 5 mg. tetrasodium 2-$^{14}$C-methyl-1 : 4-naphthohydroquinone diphosphate (Butanol/methanol system). The three peaks of activity are, respectively, the injected compound (I), and two metabolites A and B (see Table II).

It is probable that the figure found for the D.A.R. of the tumour is minimal since the whole tumour tissue contains more stroma than tumour cells. Since fluorescence studies indicated that nearly all the fluorescence arose from the tumour cells and not from the stroma (Mitchell, 1954), this suggests that the actual D.A.R. in the tumour cells must be much higher than that measured for the tumour tissue as a whole.

These results add force to earlier indications that 2-$^{14}$C-methyl-1 : 4-naphthohydroquinone bis (disodium phosphate) can concentrate in tumour tissue for short periods and give hope that a compound may be developed with a similar but more pronounced radiosensitizing effect possibly acting by virtue of selective concentration in tumour tissue.

TABLE III.—*The $^{14}C$ Content of Tissue Samples from Rats with The Walker 256 Carcinoma following Intravenous Injection of 5 to 10 mg. of $^{14}C$-Compound I.*

| | D.A.R. (wet tissue). | | | | |
|---|---|---|---|---|---|
| | 1 hour. | | 1½ hours. | 2 hours. | 24 hours. |
| Rat No. | 14/1 | 21/1 | 17/1 | 18/1 | 1/2 |
| Tumour | 0·3 | 0·24 | 0·21 | 0·095 | 0·12 |
| Muscle | 0·15 | 0·11 | 0·05 | 0·093 | 0·10 |
| Testis | 0·26 | 0·23 | 0·16 | 0·13 | 0·17 |
| Kidney | 2·0 | 2·1 | 1·1 | 0·67 | 0·46 |
| Spleen | 0·3 | 0·24 | 0·16 | 0·12 | 0·11 |
| Liver | 0·66 | 0·61 | 0·28 | 0·29 | 0·18 |
| Blood | 0·67 | 0·53 | 0·37 | 0·27 | 0·12 |
| | (4·4%) | (3·6%) | (2·4%) | (1·8%) | (0·79%) |
| Urine | — | — | 32% | — | 22% |
| Faeces | — | — | — | — | 5·3% |
| $CO_2$ | 0·01% | — | — | 0·03% | 5·9% |

Percentages in the lower half of the table indicate percentage of the injected dose associated with the blood and excreta.

TABLE IV.—*The $^{14}C$ Content of Tissue Samples from Rats with The Walker 256 Carcinoma following Intravenous Injection of 5 to 10 mg. of $^{14}C$-Compound I.*

| | D.A.R. (dry tissue). | | | | |
|---|---|---|---|---|---|
| | 1 hour. | | 1½ hours. | 2 hours. | 24 hours. |
| Tumour | 0·44 | 0·37 | 0·36 | 0·11 | 0·18 |
| Muscle | 0·13 | 0·10 | 0·06 | 0·08 | 0·16 |
| Testis | 0·43 | 0·42 | 0·27 | 0·23 | 0·28 |
| Kidney | 1·7 | 1·9 | 1·3 | 0·65 | 0·42 |
| Spleen | 0·31 | 0·22 | 0·18 | 0·13 | 0·12 |
| Liver | 0·58 | 0·45 | 0·26 | 0·23 | 0·17 |

SUMMARY

The metabolism of tetrasodium 2-$^{14}$C-methyl-1 : 4-naphthohydroquinone diphosphate has been studied in rats. About 30 per cent of the activity is excreted in the urine within 90 minutes of injection and activity is also found in respired carbon dioxide.

Measurement of the Differential Absorption Ratio of various internal organs shows that, for short periods after injection, the compound concentrates more in tumour tissue than in muscle, but that it also accumulates in organs concerned with detoxification, excretion and Vitamin K function.

The authors record their thanks to Professor J. S. Mitchell, F.R.S., for his continual interest and encouragement, and to Mr. E. A. King for his help with the animal experiments. One of us (D. R. M.) is indebted to the Medical Research Council for a research grant.

REFERENCES

ANDREWS, K. J. M., MARRIAN, D. H. AND MAXWELL, D. R.—(1956) *J. chem. Soc.*, 1844.
DOISY, E. A.—(1949) *116th Meeting of Amer. chem. Soc.*, Abstracts, p. 66.
GARTLAND, G. F. AND KOCH, F. C.—(1928) *Amer. J. Physiol.*, **85**, 540.

JAQUES, L. B., MILLAR, G. J. AND SPINKS, J. W. T.—(1954) *Schweiz. med. Wschr.*, **29**, 792.
MACKENZIE, C. G., CHANDLER, J. P., KELLER, E. B., CROSS, N. AND DU VIGNEAUD, V.—(1949) *J. biol. Chem.*, **180**, 99.
MITCHELL, J. S.—(1953) *Brit. J. Cancer*, **7**, 313.—(1954) Radiobiology Symposium, Butterworth's Scientific Publications, p. 170.
*Idem* AND SIMON-REUSS, I.—(1947) *Nature*, **160**, 98.—(1952*a*) *Brit. J. Cancer*, **6**, 305.—(1952*b*) *Ibid.*, **6**, 317.
MOORE, F. D., TOBIN, L. H. AND AUB, J. C.—(1943) *J. clin. Invest.*, **22**, 161.
MORRISON, D. C., and CROWLEY, J. F.—(1952) *Univ. Cal. Rad. Lab. Rep.*, UCRL—1759.
NEUKOMM, S.—(1954) Radiobiology Symposium, Butterworth's Scientific Publications, p. 189.
*Idem*, PEGUIRON, L., LERCH, P. AND RICHARD, M.—(1953) *Arch. int. Pharmacodyn.*, **93**, 373.
RICKERT, D. A.—(1944) *J. biol. Chem.*, **154**, 1.—(1951) *Ibid.*, **189**, **763**.
SOLVONUK, P. F., JAQUES, L. B., LEDDY, J. E., TREVOY, Z. W. AND SPINKS, J. W. T.—(1952) *Proc. Soc. exp. Biol., N.Y.*, **79**, 597.
TAYLOR, D. AND SHARPE, J.—(1951) *Proc. Instn. elect. Engrs.*, **98**, Pt. 2, 174.
VAN SLYKE, D. V.—(1954) *Analyt. Chem.*, **26**, 1706.
WICK, A. N., BARNET, H. W. AND ACKERMAN, N.—(1949) *Ibid.*, **21**, 151.